# DUSP3 regulates phosphorylation-mediated degradation of occludin and is required for maintaining epithelial tight junction

**DOI:** 10.1186/s12929-022-00826-x

**Published:** 2022-06-15

**Authors:** Hsiao-Chin Chou, Chun-Mei Cheng, Chi-Hwa Yang, Tzu-Yin Lin, Ya-Wen Liu, Tse-Hua Tan, Yi-Rong Chen

**Affiliations:** 1 Institute of Molecular and Genomic Medicine, National Health Research Institutes, Zhunan, 35053 Taiwan; 2 Immunology Research Center, National Health Research Institutes, Zhunan, Taiwan; 3 Department of Pathology & Immunology, Baylor College of Medicine, Houston, TX USA

**Keywords:** DUSP3/VHR, Epithelium, FAK, Occludin, Tight junction

## Abstract

**Background:**

Tight junctions (TJ) are multi-protein complexes that hold epithelial cells together and form structural and functional barriers for maintaining proper biological activities. Dual specificity phosphatase 3 (DUSP3), a suppressor of multiple protein tyrosine (Tyr) kinases, is decreased in lung cancer tissues. Here we demonstrated the role of DUSP3 in regulation of epithelial TJ.

**Methods:**

Barrier functions of TJ were examined in wild-type or DUSP3-deficient lung epithelial cells. Animal and clinical data were analyzed for the association between DUSP3 deficiency and lung cancer progression. Proximity ligation assay, immunoblotting, and phosphatase assay were performed to study the effect of DUSP3 on the TJ protein occludin (OCLN). Mutations of Tyr residues on OCLN showed the role of Tyr phosphorylation in regulating OCLN.

**Results:**

Compared to those of the DUSP3-expressing cells, we found the expression and distribution of ZO-1, a TJ-anchoring molecule, were abnormal in DUSP3-deficient cells. OCLN had an increased phosphorylation level in DUSP3-deficient cells. We identified that OCLN is a direct substrate of DUSP3. DUSP3 regulated OCLN ubiquitination and degradation through decreasing OCLN tyrosine phosphorylation directly or through suppressing focal adhesion kinase, the OCLN kinase.

**Conclusion:**

Our study revealed that DUSP3 is an important TJ regulatory protein and its decrease may be involved in progression of epithelial cancers.

**Supplementary Information:**

The online version contains supplementary material available at 10.1186/s12929-022-00826-x.

## Background

Normal epithelial cells, particularly in simple epithelium, have apical and basal-lateral polarity and form barrier against environmental challenges. This function depends on normal cell–cell interaction apparatuses, including adherens junction (AJ), desmosome, as well as gap and tight junctions (GJ and TJ) [1, 2]. During oncogenesis, cancer cells undergo epithelial-mesenchymal transition (EMT), lose their polarity, and gain higher migratory and invasive abilities. In these processes, cancer cells usually have dysfunctional cell–cell junctions, especially in AJ and TJ [3, 4]. During EMT, the strong AJs formed by E-cadherin are replaced by weak N-cadherin interaction, allowing epithelial cells to escape from the homotypic interactions that tie them to adjacent cells. Re-expression of E-cadherin usually suppresses oncogenicity [3]. On the other hand, TJs are specifically important for maintaining epithelial barrier because of their structure [4]. TJs, tightly fused regions between the outer membrane of adjacent cells, are formed by integral transmembrane proteins such as OCLN and claudins, anchoring proteins such as zonula occludens (ZO)1–3, and other TJ-associated/regulatory proteins [4, 5]. The barrier property of TJ is a natural obstacle for cancer cells to break away from their neighboring cells; therefore, abnormalities of TJ are frequently observed in epithelial cancers [4]. However, cell–cell junctions do not always negatively regulate cancer development. Cell–cell interaction has been shown to coordinate collective cell migration, which is a critical process in cancer metastasis [6]. Therefore, the regulation of cell–cell junctions is a dynamic process during cancer progression, and the regulatory mechanisms are warranted for further studies.

Dual-specificity phosphatases (DUSPs) are initially identified as suppressors of mitogen-activated protein kinases (MAPKs) and are also named MKPs (MAPK phosphatases) [7–9]. However, many lately identified DUSPs have little or no phosphatase activity against MAPKs [10–14]. These novel DUSPs have smaller sizes and lack the MAPK-binding domain, in comparison to MKPs. They are later classified as atypical DUSPs [7–9, 15]. Many studies reveal that atypical DUSPs are important in regulating tyrosine kinase signaling and many cellular processes [13, 15–20]. DUSP3/VHR, related to the *vaccinia* virus phosphatase VH1, is initially found to be a phosphatase that dephosphorylates multiple protein tyrosine kinases (PTKs) in vitro [21]. Later reports show that DUSP3 suppresses activation of MAPKs in different systems [22–24]. However, DUSP3 is a weak phosphatase against MAPKs in comparison to other MKPs [18, 25]. On the other hand, DUSP3 dephosphorylates Stat5 and inhibits Stat5 activation by interferons [26]. DUSP3 also dephosphorylates epidermal growth factor receptor (EGFR), suppresses tumor formation by lung cancer cells, and its expression levels are decreased in lung cancer tissues [18]. DUSP3-null mice are viable but their endothelial cells fail to form angiogenic tubes and their platelets also show defects in activation and thrombus formation [27, 28]. DUSP3-deficient mice also have increases in M2-like macrophages and show tolerance to lipopolysaccharide-induced endotoxin shock and microbial septic shock [29]. The molecular mechanisms involved in these phenotypes are not clear. We find that DUSP3 is a direct suppressor of focal adhesion kinase (FAK), and that DUSP3-deficient cells have abnormalities in cell adhesion and migration [30].

In this study, we found that DUSP3-deficient cells had defective TJ and barrier functions. DUSP3 was a phosphatase that dephosphorylated OCLN and regulated OCLN ubiquitination and degradation. Our data implicated the loss of DUSP3 expression in lung cancer may participate in the tumorigenic process.

## Methods

### Experimental animals and cell cultures

DUSP3-knockout mice and EGFR-Del (E746-A750) transgenic (Tg) mouse (line A) as well as the genotyping methods of these animals were reported previously [30, 31]. All experimental procedures using animals were approved by the Institutional Animal Care and Use Committee of National Health Research Institutes (NHRI) and animal care was in accordance with institution guidelines. DelA cell lines with various DUSP3 status were established and cultured as previously described [30]. H1299-DUSP3_TR_ and H1299-DUSP3-CS_TR_ cells for inducible expression of DUSP3 (wild-type or mutant) were described previously [30]. H1299-DUSP3_TR_/OCLN and H1299-DUSP3-CS_TR_/OCLN cells were established by transfecting a Flag-tagged OCLN vector into their parental cell line, respectively, followed by two weeks of G418 selection (1.5 mg/ml). H1299 cells expressing various OCLN mutants were established similarly by permanent expression of the respective OCLN vectors. All H1299 derivatives were cultured in RPMI-1640 supplemented with 10% fetal calf serum plus penicillin and streptomycin.

### Plasmid constructions

The coding sequence of human OCLN was PCR-amplified using a H1299 cDNA pool as the template. The PCR primers are OCLN-Forward (F), 5’-GCGGAGCTCATGTCATCCAGGCCTCTTGAAAG-3’; OCLN-Reverse (R), 5’-GCGAAGCTTCTATGTTTTCTGTCTATCATAG-3’. The OCLN-coding fragment was digested by restriction enzymes and was inserted into the pCMV-Tag2C between Srf I and Hind III sites to form the expression vector of Flag-tagged OCLN. Potential human OCLN Tyr phosphorylation sites were identified through the PhosphoSitePlus (PSP) program [32, 33]. Tyr residues with more records in previous high throughput papers than Tyr 398/402 were selected for site-directed mutagenesis. All Tyr (Y) to Phe (F) mutants were constructed from the Flag-OCLN vector using the site-direct mutagenesis method. The Flag-OCLN-Y398/402F mutant vector was constructed as described previously by Elias et al. [34]. All other OCLN mutant vectors were constructed using primers as described below.

Y287F-F, 5’-CAAGGAACACATTTTTGATGAGCAGCC;

Y287F-R, 5’-GGCTGCTCATCAAAAATGTGTTCCTTG;

Y315F-F, 5’-CCCATCTGACTTTGTGGAAAGAGTTGAC;

Y315F-R, 5’-GTCAACTCTTTCCACAAAGTCAGATGGG;

Y325F-F, 5’-GTCCCATGGCATTCTCTTCCAATGG;

Y325F-R, 5’-CCATTGGAAGAGAATGCCATGGGAC;

Y443F-F, 5’-GGCCTACAGGAATTCAAGAGCTTACAATC;

Y443F-F, 5’-GGCCTACAGGAATTCAAGAGCTTACAATC;

Y443F-R, 5’-GATTGTAAGCTCTTGAATTCCTGTAGGCC.

### Antibodies and reagents

The anti-Myc monoclonal antibody was prepared from a hybridoma (clone 9E10). Anti-Flag monoclonal antibody (M2), M2-conjugated agarose beads, and anti-β-actin antibody were purchased from Sigma-Aldrich (St. Louis, MO, USA). The anti-DUSP3 (sc-374161), anti-FAK (sc-1688), anti-OCLN (H-279; sc-5562), anti-Src (sc-5266) and peroxidase-conjugated secondary antibodies were purchased from Santa Cruz (Santa Cruz, CA, USA). The anti-ZO-1 (40–2200, 61–7300 and 33–9100), anti-OCLN (33–1500) antibodies were obtained from ThermoFisher Scientific (Pittsburgh, PA, USA). The anti-pFAK (Y397; ab81298) antibody was purchased from Abcam (Cambridge, UK). The anti-phospho-tyrosine antibodies 4G10 was purchased from Upstate Biotechnology (Waltham, MA, USA). The anti-pSrc (Tyr416; #2113), anti-pSrc (Tyr527; #2105), anti-DUSP3 (#4752) and anti-phospho-Tyr (pTyr1000) antibodies were purchased from Cell Signaling Technology (Beverly, MA, USA). Anti-pan-cadherin (GTX132646), anti-OCLN (GTX114949) and anti-ITCH (GTX02845) antibodies were obtained from GeneTex Inc (Hsinchu, Taiwan). The anti-DUSP3 antibody (AP8478a) was purchased from Abgent (San Diego, CA, USA). G418, cycloheximide, and H_2_O_2_ were purchased from Sigma-Aldrich (St. Louis, MO, USA). FAK inhibitor defactinib was purchased from ThermoFisher Scientific (Pittsburgh, PA, USA).

### Cell and tissue extract preparation

Whole cell extract was prepared by suspending cultured cells in lysis buffer (50 mM Tris [pH 8.0], 150 mM NaCl, 1% Triton-X-100, 0.5% deoxycholate, 0.1% SDS, 2 µg/ml leupeptin, 5 µg/ml aprotinin, 1 mM PMSF, 1 mM dithiothreitol (DTT), and 1 mM Na_3_VO_4_). The cell extracts were kept on ice with vigorous mixing four times with 5 min intervals. The extracts were cleared by centrifugation at 14,000*g* for 10 min, and the supernatants were collected for subsequent analyses or stored at − 80 °C. Mouse tissue extracts were prepared by homogenizing the tissue fragments in lysis buffer using the MagNA Lyser Green Beads protocol (Roche Diagnostics, Indianapolis, IN, USA) and stored in − 80 °C for further characterization.

### Immunoprecipitation (IP) and phosphatase assay

Target proteins from cell extracts were precipitated by a specific antibody (1–2 μg) plus protein A/G-agarose beads in 1.5 ml of lysis buffer with continuously rotation at 4 °C for 2 h. All samples for detection of protein interaction (co-IP) were prepared and processed in lysis buffer without deoxycholate and SDS. The IP complexes were washed three times with lysis buffer. Samples (various OCLN proteins) for enzymatic assays were washed two more times with phosphatase reaction buffer (50 mM Tris [pH 7.0], 50 mM Bis–Tris, 100 mM sodium acetate, and 10 mM DTT) or kinase buffer (20 mM MOPS, pH 7.6, 2 mM EGTA, 10 mM MgCl_2_, 1 mM DTT, 0.1% Triton X-100, and 1 mM Na_3_VO_4_), and the substrates were eluted with respective reaction buffer containing the Flag peptide (200 μg/ml). Aliquots of eluted proteins were mixed with 5–20 µg/ml of GST-DUSP3 or GST-DUSP3(C124S) in 100 µl of phosphatase reaction buffer. The phosphatase reaction was performed at 37 °C for 1 h. The enzymatic reactions were terminated by adding SDS sample buffer and heating at 95 °C for 5 min. The samples were separated by SDS-PAGE and subjected to immunoblot analyses.

### Permeability and trans-epithelial electric resistance (TEER) assays

DelA cells were seeded in the 0.4-μm transwell (Corning, Kennebunk, ME, USA) and cultured at the regular condition until cells reached confluence. FITC-dextran (molecular weight 3000–5000; Sigma-Aldrich) was added into upper chamber to a final concentration 25 μg/ml. After another 2 h in culture containing FITC-dextran, 100 μl medium was extracted from bottom layer for FITC detection. The excitation and emission wavelength for measurement were 490 and 520 nm, respectively. The FITC-dextran flux measured in the absence of cells was assigned as 100%. The TEER assays were performed using an Millicell ERS-2 epithelial tissue volt/ohm meter (Merck Millipore, Burlington, MA, USA). The 0.4-μm transwell inserts, with effective membrane area of 0.3 cm^2^, were seeded with or without DelA derivative cells. The TEER was measured right after cell seeding and after cell confluence. The basolateral probe inserted into medium of bottom chamber and the apical probe in the upper insert. The resistance readings on the meter were recorded as the TEER of cells.

### Proximity ligation assay (PLA)

The assay was performed using the Duolink in Situ kit according to the manufacture’s instruction (Olink Bioscience, Uppsala, Sweden) [35]. DelA-F13 or F16R cells were seeded onto 3-cm glass-bottom dishes (ibidi; Martinsried, Germany) and were fixed with 4% paraformaldehyde for 20 min, permeabilized by 0.1% Triton X-100 for 5 min at room temperature, and then blocked with Duolink II Blocking Solution (Olink Bioscience) for 30 min at 37** °C**. Primary antibodies were diluted (1:100) in the Antibody Diluent (Olink Bioscience), and the samples were incubated at 4** °C** overnight. After the removal of primary antibodies and washing with buffer A (Olink Bioscience), mixture of two probes (anti-rabbit PLUS and anti-mouse MINUS) was added, and the samples were incubated for 1 h at 37** °C**. Ligation and amplification were proceeded at 37** °C** for 30 min and 2 h, respectively. Finally, cell nuclei were stained with DAPI, and the PLA signals were analyzed by a Leica TCS SP5 confocal microscope (Mannheim, Germany). Primary antibodies used in this study included anti-DUSP3 (clone F2, sc-374161), anti-OCLN (H-279; sc-5562), and anti-ZO-1 (40–2200) antibodies.

### Immunofluorescence (IF) staining, immunoblots, and Phos-tag SDS-PAGE analysis

IF staining and standard immunoblotting assays were performed as described previously [17]. Phos-tag SDS-PAGE analysis was performed according to the manufacturer instruction (Wako Pure Chemical Industry, Japan) [36]. In brief, 25 μM of Phos-tag reagent and MnCl_2_ were thoroughly mixed into the stacking gel solution before polymerization. Before transferring the proteins onto PVDF membranes, the SDS-PAGE gels were incubated in 15 ml of transfer buffer containing EDTA (100 mM) with gentle shaking for 10 min three times followed by one incubation with transfer buffer without EDTA. All the other processes were performed as the standard SDS-PAGE protocol. The presented immunoblot images are representative data from at least three independent experiments.

### Survival analysis of lung adenocarcinoma (LAC) patients

The data of *DUSP3* expression levels (RNA sequencing) and survival of LAC patients were acquired from The Cancer Genome Atlas (TCGA) through the Kaplan Meier plotter [37, 38]. LAC dataset was selected among the Pan-Cancer RNA-seq program to examine the correlation between *DUSP3* expression and overall patient survival (up to 120 months). The data was analyzed with an automatically selected best cutoff.

## Results

### DUSP3-deficient cells have defective TJ and barrier function

**Fig. 1:**
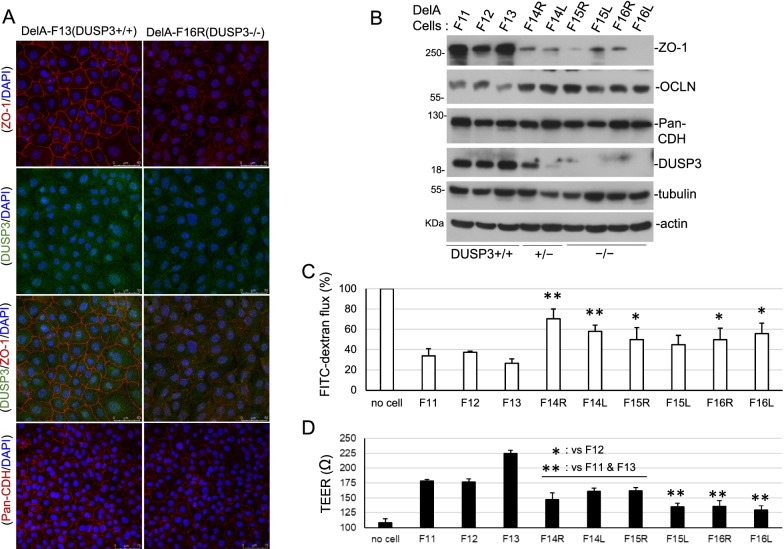
DUSP3-deficient cells have defective TJ and barrier function. **A** Confluent DelA-F13 cells (DUSP3 +/+) and F16R cells (DUSP3−/−) were subjected to immunofluorescence (IF) staining using anti-ZO-1 (40–2200) and anti-DUSP3 (sc-374161) antibodies or an anti-pan-cadherin (CDH) antibody. DAPI was used for nuclear staining. **B** Cell lysates collected from DelA cell lines with different DUSP3 status were subjected to immunoblot analyses using indicated antibodies. **C** Confluent DelA cells were subjected to permeability assays against FITC-Dextran. The Student t-tests were performed between individual DUSP3-deficient cell lines and the mean value of three DUSP3 +/+ cell lines. **D** Trans-epithelial electric resistance (TEER) was measured in DelA cells after reaching confluence. The Student t-tests were performed between any individual DUSP3-deficient cell lines and DUSP3 +/+ cell lines. The data presented were the mean and standard deviations (*p < 0.05; **p < 0.01) of the triplicated experiments (**C** and **D**)

Previously we have established mouse lung epithelial cell lines (DelA series) with different DUSP3 status [30]. We noticed that DUSP3-deficient cells (DUSP3 +/– and DUSP3−/−) failed to have close contacts between cells that were observed in DUSP3-wild type cells upon confluence. We had examined the distribution patterns of various cell–cell junction molecules and found that ZO-1, a TJ protein, was significantly decreased and did not show the typical TJ distribution in DUSP3−/− cells in comparison with DUSP3 +/+ cells (Fig. 1A). IF staining with a different pair of ZO-1 and DUSP3 antibodies showed a similar ZO-1 distribution profile (Additional file 1: Fig. S1, Additional file 1). On the contrary, pan-cadherin immunofluorescence (IF) staining showed similar patterns between DUSP3−/− and DUSP3 +/+ cells (Fig. 1A). We also found a significant decrease of ZO-1, but not OCLN and cadherin proteins, in DUSP3 +/– and DUSP3−/− cells through immunoblotting assays (Fig. 1B). Except DelA-F15L cells (*p* = 0.059), other DUSP3 +/– and DUSP3−/− cells exhibited significantly higher permeability to FITC-dextran particles (Fig. 1C). Also, DUSP3 +/– and DUSP3−/− cells showed lower trans-epithelial electric resistance (TEER) in comparison to that in DUSP3 +/+ cells (Fig. 1D). The results of both assays showed that DUSP3-deficient lung epithelial cells had defective barrier functions.

Some wild type and DUSP3-deficient DelA derivatives (DelA-F12, F15R, F15L, F16L) showed apparent G2/M arrest upon confluence, but this characteristic was not correlated with the DUSP3 deficiency. Also, no increases in Sub-G1 staining population (apoptotic cells) were observed in DUSP3-deficient cells in comparison to wild type cells (Additional file 1: Fig. S2, Additional file 1). Therefore, the defective TJ and barrier function may not be the results of cell cycle arrest or apoptosis. Interestingly, we observed DNA hyperploidy (F14L, F15L, and F16R) or hypoploidy (F15R) in DUSP3-deficient cells in comparison to the DUSP3 +/+ cells, suggesting an abnormality in maintaining chromosomal stability (Additional file 1: Fig. S2, Additional file 1).

### DUSP3-deficient mice have decreased ZO-1 expression and a faster progression of EGFR mutant-driven lung adenocarcinoma

**Fig. 2:**
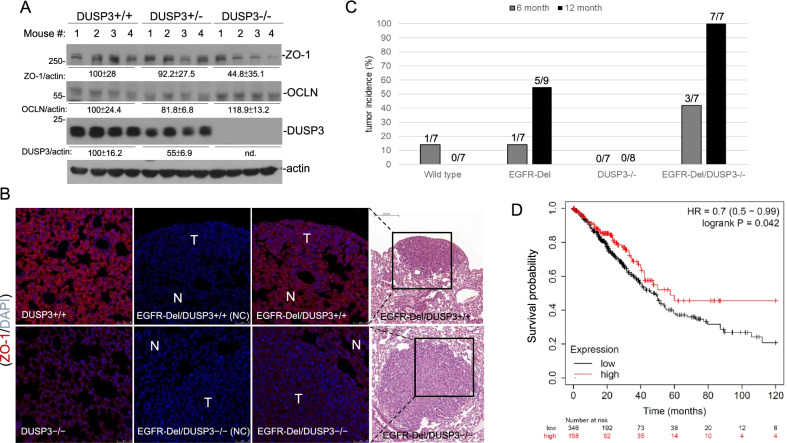
DUSP3-deficient mice have decreased ZO-1 expression and a faster progression of EGFR mutant-driven lung adenocarcinoma. **A** Lung tissues derived from mice of different DUSP3 status were subjected to extract preparation and immunoblot analyses of proteins as indicated. The numbers showed the means and standard deviations of the signals. The means in DUSP3 +/+ mice were assigned as 100. **B** Lung tissue sections derived from DUSP3 +/+ and DUSP3−/− mice with or without an EGFR-Del transgenic gene were subjected to IF staining using a ZO-1 antibody. T and N indicated tumor and adjacent normal tissues, respectively. Hematoxylin and Eosin (H&E) staining of the consecutive sections showed the locations of the tumors. NC: negative control staining without the primary antibody. **C** Tumor incidences in 6-M and 12-M old male mice with indicated genotypes were analyzed by pathological examination of lung sections staining with hematoxylin and eosin. **D** The correlation between DUSP3 expression (RNA sequencing data) and LAC patient survival in the TCGA database was analyzed through the Kaplan Meier Plotter

To verify the results in cultured cells, we found that ZO-1 levels were also significantly lower in lung tissues of DUSP3−/− mice (*p* < 0.05), compared with those in wild type mice (Fig. 2A). However, other than minor increases in cellular density, we did not observe apparent histological changes in DUSP3−/− lung tissues (Additional file 1: Fig. S3, Additional file 1). Because of the frequent loss of TJ in transformation of epithelial tissues, we interbred DUSP3-knockout mice with EGFR-Del transgenic (Tg) mice and examined the effect of DUSP3 deficiency in lung adenocarcinoma (LAC) progression. Immunofluorescence staining (IF) assays showed a significantly decrease of ZO-1 in the lung tissue of DUSP3−/− mice, although with a similar distribution pattern, in comparison to that in DUSP3 +/+ tissue (Fig. 2B). Tumors developed in EGFR-Del Tg/DUSP3−/− mice displayed even lower ZO-1 staining compared with the adjacent normal tissues (Fig. 2B). In contrast, tumors developed in EGFR-Del-Tg/DUSP3 +/+ mice showed similar levels of ZO-1 as the adjacent lung tissue, although with an abnormal intracellular distribution (Fig. 2B). This data indicated that DUSP3 ablation exacerbated TJ defects in LAC, which might promote tumor progression.

Previously we showed that DUSP3 expression was decreased in non-small cell lung cancer tissues [18]. We have studied the tumor-promoting ability of DUSP3 deficiency by examining the onset of LAC in DUSP3 +/+ and DUSP3−/− mice with or without the EGFR-Del Tg gene. As shown in Fig. 2C, the DUSP3 loss alone did not increase the incidence of LAC development in mice; however, the development of LAC was significantly faster in EGFR-Del-Tg mice combined with the loss of the DUSP3 gene. Consistent with the observation in experimental animals, we found that low DUSP3 expression levels were associated with worse survival outcomes in LAC patients through analyzing the clinical data in the TCGA database (Fig. 2D). The collective data implicated a novel association between loss of DUSP3 expression with defective TJ and epithelial cancer progression.

### DUSP3 deficiency associates with an increase of OCLN phosphorylation

**Fig. 3:**
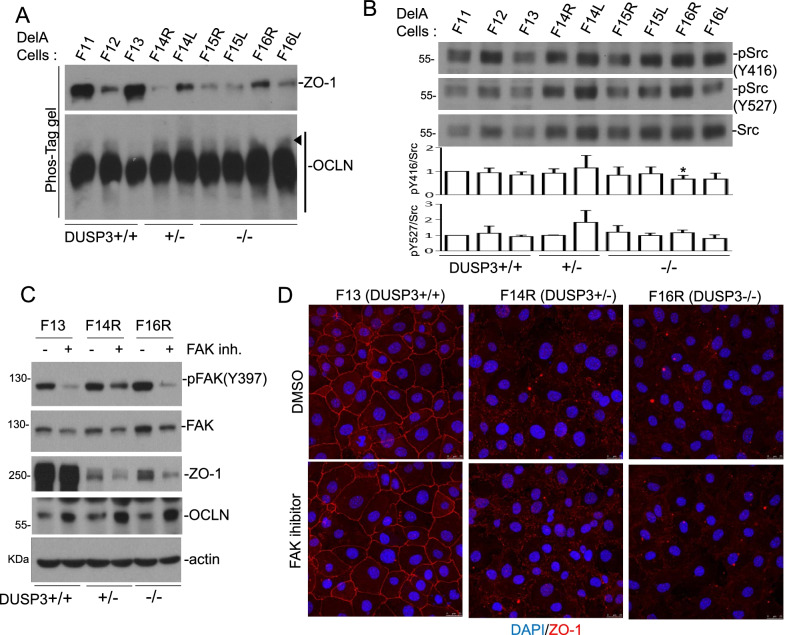
DUSP3-deficiency is associated with increased OCLN phosphorylation in DelA cell lines. **A** Cell lysates collected from various DelA cell lines were subjected to 7% SDS-PAGE analyses containing 25 μM of Phos-Tag reagent plus MnCl_2_, followed by immunoblot analyses using indicated antibodies. Phosphorylated OCLN with slower mobility was indicated by an arrowhead. **B** The expression and phosphorylation levels of Src were analyzed by immunoblot assays. The bar graphs showed the means and standard deviations of three independent experiments. The value of DelA-F11 was assigned as 1. * p < 0.05. **C** and **D** Confluent DelA-F13, F14R and F16R cells were subjected to a 24 h treatment of an FAK inhibitor (Defactinib; 5 μM) and the cells were collected and analyzed by immunoblot assays (**C**) or IF staining (**D**) using indicated antibodies

DUSP3 is a phosphatase with substrate preference on phospho-tyrosine (pTyr/pY) [39–41]. Tyr phosphorylations of ZO-1 and OCLN have both been implicated in the modulation of TJ [34, 42]; therefore, we first examined the possible modulation of phosphorylation in ZO-1 and OCLN in DUSP3-deficient DelA cells using the Phos-tag SDS-PAGE analysis. In comparison to that in DUSP3 +/+ cells, an increase of phosphorylated OCLN with slower mobility, but not ZO-1, was observed in DUSP3 +/– and DUSP3−/− cells (Fig. 3A). Both Src and FAK have been implicated in the phosphorylation of OCLN [34, 42]. We did not observe a consistent change in the activating and inhibitory Tyr phosphorylations (Y416 and Y527, respectively) of Src kinase in DUSP3-deficient cells (Fig. 3B); this was in agreement with our previous finding that DUSP3 did not regulate Src directly [18]. On the contrary, DUSP3 is a phosphatase targeting FAK and pFAK levels are increased at focal adhesions of DUSP3−/− cells [30]. We found that defactinib, an FAK inhibitor, increased OCLN levels in wild type and DUSP3-deficient cells (Fig. 3C). Defactinib treatment further decreased the levels of ZO-1 and did not restore the TJ staining pattern of ZO-1 in DUSP3-deficient cells (Fig. 3C,D). These data indicated that FAK had manifold impacts on TJ proteins and might have a negative effect on OCLN expression.

### DUSP3 associates with and de-phosphorylates OCLN

**Fig. 4:**
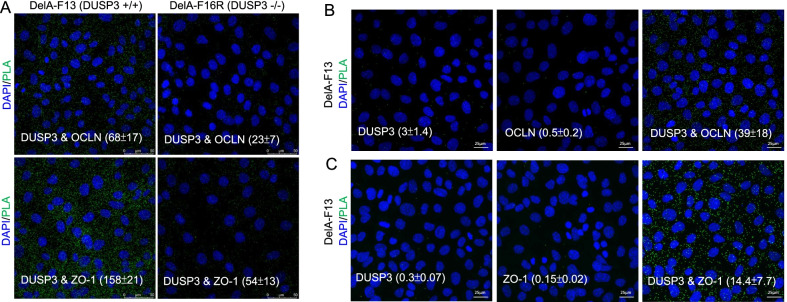
DUSP3 is associated with ZO-1 and OCLN in a cellular context. **A** DelA-F13 and F16R cells were subjected to the PLA to examine the close proximity of DUSP3 with OCLN or ZO-1 in the cellular context. **B** and **C** PLAs with the presence of only one primary antibody (DUSP3, OCLN, or ZO-1) was performed as the negative control experiments. DAPI was used for nuclear staining. Quantitative numbers were the mean and standard deviations of PLA-positive signals per cells from at least 200 cells

We have examined the physical interaction between DUSP3 and OCLN as well as ZO-1 and did not observe the binding between DUSP3 and these two proteins using co-immunoprecipitation (co-IP) assays. Nevertheless, we found that these proteins were in close proximity (within 40 nm) in a cellular context using the PLA. The PLA signals were significantly higher (*p* < 0.01) in DelA-F13 cells than those in DUSP3−/− DelA-F16R cells (Fig. 4A). As the negative controls, assays with single primary antibody (DUSP3, OCLN, or ZO-1) did not produce significant PLA signals (Figs. 4B, C). This result suggested that DUSP3 and OCLN, as well as DUSP3 and ZO-1 were in a protein complex even though their interaction was not strong enough to be detected by the conventional co-IP method.

**Fig. 5:**
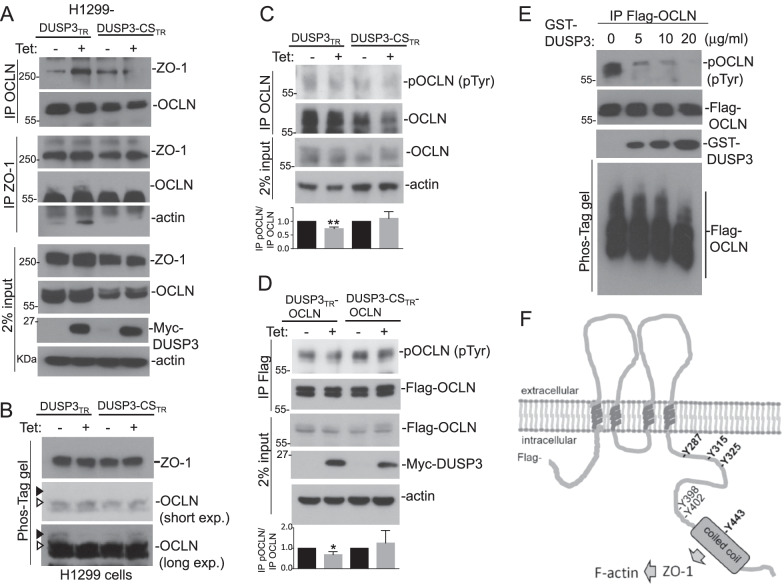
DUSP3 is an OCLN phosphatase. **A** H1299-DUSP3_TR_ and H1299-DUSP3-CS_TR_ cells were treated with or without tetracycline (Tet; 1 μg/ml) for 8 h. OCLN and ZO-1 proteins were immunoprecipitated and the immunocomplexes were examined for the presence of indicated proteins. Two percent of extract inputs were also examined for the relative amounts of individual proteins. **B** and **C** H1299-DUSP3_TR_ and H1299-DUSP3-CS_TR_ cells were treated as in (**A**). The cell extracts were subjected to Phos-Tag SDS-PAGE assay (**B**) or to immunoprecipitation of OCLN (**C**). The samples were examined by immunoblot analyses using indicated antibodies. Arrowheads indicated the positions of phosphorylated OCLN with slower mobilities. **D** H1299-DUSP3_TR_/OCLN and H1299-DUSP3-CS_TR_/OCLN cells were treated as in (**A**). The cell extracts were subjected to anti-Flag immunoprecipitation and the samples were examined by immunoblot analyses using indicated antibodies. The bar graphs showed the means and standard deviations of three independent experiments (**C** and **D**). The value of cells without tetracycline induction was assigned as 1. *p < 0.05. **E** H1299-OCLN cells were treated with H_2_O_2_ (1 mM) for 30 min and phosphorylated OCLN were affinity-purified and subjected to in vitro DUSP3 phosphatase reactions. **F** Schematic illustration of OCLN showed the major Tyr(Y) phosphorylation sites documented in the PhosphoSitePlus post-translational modification (PTM) database. The coiled-coil domain of OCLN was indicated with a grey box. All the OCLN constructs used in this study were Flag-tagged at the N termini

The defective barrier function in DUSP3−/− cells and the association between DUSP3 and OCLN (and ZO-1) suggested that DUSP3 was involved in the regulation of TJ. We found that the inducible expression of DUSP3 in H1299 cells augmented the interaction between OCLN and ZO-1 (Fig. 5A). In contrast, expression of a DUSP3 phosphatase-dead mutant (DUSP3-CS) decreased the interaction between OCLN and ZO-1 (Fig. 5A). Interestingly, DUSP3 expression also increased the interaction between ZO-1 and cytoskeleton β-actin (Fig. 5A).

Using Phos-tag SDS-PAGE, we found that the inducible expression of DUSP3 decreased the level of OCLN with slow mobility whereas DUSP3-CS increased it, suggesting that DUSP3 inhibited OCLN phosphorylation (Fig. 5B). By measuring the Tyr phosphorylation levels of immunoprecipitated OCLN (both endogenous OCLN and exogenous Flag-OCLN), we also found that expression of DUSP3, but not DUSP3-CS, decreased the Tyr phosphorylation of OCLN (Fig. 5C, D). In addition, recombinant GST-DUSP3 directly removed Tyr phosphorylation from OCLN in vitro (Fig. 5E). The collective results supported that DUSP3 dephosphorylated OCLN directly and regulated its functions.

Tyr phosphorylation of OCLN has been implicated in regulating OCLN degradation, and Src phosphorylation on Y398 and Y402 residues of OCLN has been shown to decrease OCLN-ZO-1 interaction [34, 43]. Accumulated data of previous proteomic analyses summarized in the PSP server show that OCLN is phosphorylated on numerous Tyr residues at its carboxyl terminus (Fig. 5F). It was intriguing to know the mechanism by which DUSP3 regulated OCLN through modulating its phosphorylation.

### DUSP3 targets multiple Tyr residues at the carboxyl-terminus of OCLN

**Fig. 6:**
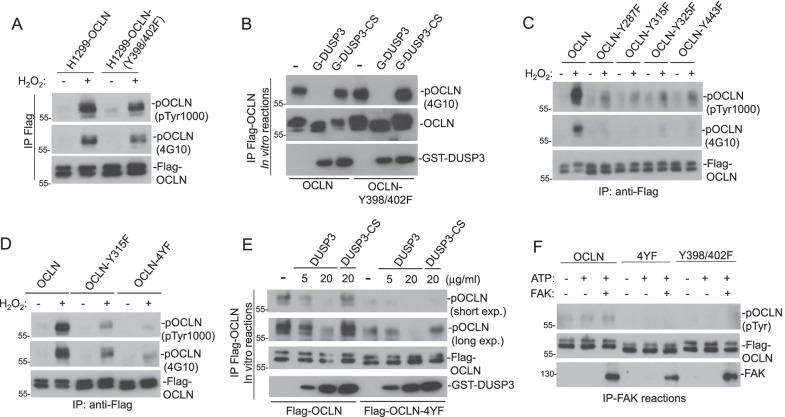
DUSP3 targets multiple Tyr residues at the carboxyl-terminus of OCLN. **A** and **B** H1299 cells expressing with wild-type OCLN or OCLN mutant (OCLN-Y398/402F) were treated with or without H_2_O_2_ (1 mM) for 30 min. Wild-type and mutated OCLN proteins were immunoprecipitated and analyzed for their Tyr-phosphorylation levels (**A**) or were subjected to in vitro DUSP3 phosphatase reactions and the subsequent immunoblot analyses (**B**). **C** H1299-OCLN cells or H1299 cells-expressing indicated individual OCLN mutants were treated with or without H_2_O_2_ (1 mM) for 30 min. Wild type and mutated OCLN proteins were immunoprecipitated and analyzed for their Tyr-phosphorylation levels. **D** and **E** H1299-OCLN, OCLN (Y315F), and OCLN (Y287/315/325/443F; named 4YF) cells were treated with or without H_2_O_2_ (1 mM) for 30 min. Wild-type and mutated OCLN proteins were immunoprecipitated and analyzed for their Tyr-phosphorylation levels (**D**) or were subjected to in vitro DUSP3 phosphatase reactions (**E**). **F** Affinity purified Flag-OCLN, OCLN-4YF, and OCLN (Y398/402F) proteins were subjected to in vitro FAK phosphorylation

Recombinant DUSP3 removed Tyr phosphorylation of OCLN completely in the in vitro phosphatase assay (Fig. 5E). To identify the Tyr residues involved in OCLN phosphorylation, we generated OCLN mutations on various Tyr residues and tested their phosphorylation susceptibilities in response to oxidative stress. First, we found that OCLN-Y398/402F mutant retained most of its Tyr phosphorylation upon H_2_O_2_ treatment (Fig. 6A). OCLN-Y398/402F could also be dephosphorylated efficiently, similar to wild type OCLN, by DUSP3 in vitro (Fig. 6B). These results indicated that Y398 and Y402, as documented in the PSP record, were not the major phosphorylation sites in OCLN; therefore, they are not the major target residues for DUSP3.

Based on the PSP data, we generated OCLN constructs with Tyr to Phe (Y-F) mutation on major Tyr phosphorylation sites including Y287, Y315, Y325, and Y443 residues. Unexpectedly, we found that all of the single Y-F mutation significantly decreased the Tyr phosphorylation level of OCLN in response to oxidative stress (Fig. 6C). The combination of all four Y-F mutations (Y287/315/325/443F; here named 4YF) further reduced the Tyr phosphorylation in OCLN in comparison to that of individual mutations (Fig. 6D). The weak Tyr phosphorylation remained in OCLN-4YF could still be removed by DUSP3 in vitro*,* but it was less efficiently than that of wild-type OCLN (Fig. 6E). Interestingly, the OCLN-4YF mutant appeared to be refractory to FAK phosphorylation in comparison with wild-type OCLN protein (Fig. 6F). These data suggested that DUSP3 and FAK might counteract each other through regulating OCLN Tyr phosphorylation.

### Counteracting effects of DUSP3 and FAK regulate OCLN ubiquitination and degradation

**Fig. 7:**
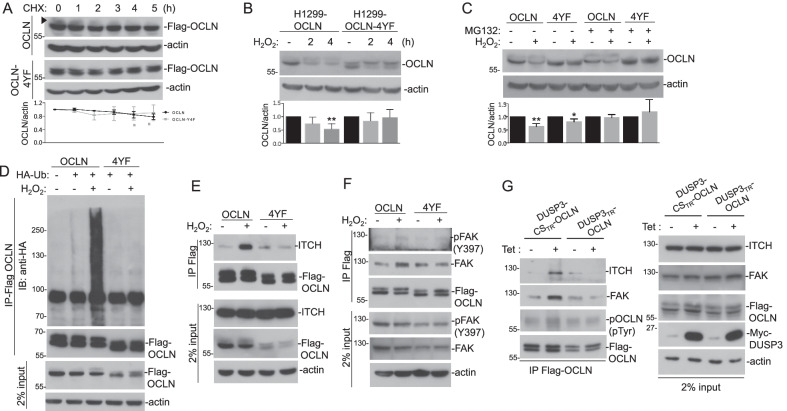
Counteracting effects of DUSP3 and FAK regulates OCLN ubiquitination and degradation. **A** H1299-OCLN and OCLN-4YF cells were treated with cycloheximide (50 μg/ml) for indicated time periods and the cell lysates were subjected to immunoblot analyses. The arrowhead indicated the position of OCLN with slower mobility. **B** H1299-OCLN and OCLN-4YF cells were pre-treated with cycloheximide (50 μg/ml) for 30 min and then were treated with H_2_O_2_ (1 mM) for indicated periods. The cell extracts were then collected and subjected to immunoblot analyses. **C** H1299-OCLN and OCLN-4YF cells were pre-treated with MG132 (0 or 50 mM) for 1 h and then were treated with or without H_2_O_2_ (1 mM) for 1 h. The cell extracts were collected and subjected to immunoblot analyses. The graphs under the immunoblots showed the mean and standard deviations of three independent experiments (A-C). * *p* < 0.05, ** *p* < 0.01. **D** Cell extracts prepared from HA-ubiquitin (HA-Ub)-transfected H1299-OCLN and OCLN-4YF were subjected to immunoprecipitation of Flag-OCLN, and the immunocomplexes were then analyzed for the presence of indicated proteins. **E** and **F** H1299-OCLN and OCLN-4YF cells were treated with or without H_2_O_2_ (1 mM) for 30 min and were subjected to extract preparation. Flag-tagged OCLN and OCLN-4YF were immunoprecipitated and the immunocomplexes were then analyzed for the presence of indicated proteins. **G** H1299-DUSP3_TR_/OCLN and H1299-DUSP3-CS_TR_/OCLN cells were treated with or without Tet (1 mg/ml) for 12 h and then were treated with H_2_O_2_ (0.5 mM) for 30 min. Cell extracts were prepared and Flag-tagged OCLN and OCLN-4YF were immunoprecipitated. The immunocomplexes were then analyzed for the presence of indicated proteins. Cell extracts (2% percent input) were also examined for the relative amounts of individual proteins

We noticed that OCLN protein level was mildly decreased upon cycloheximide (CHX) treatment, this effect was more evident in the OCLN with slower mobility, potentially the phosphorylated OCLN (Fig. 7A). In contrast, CHX failed to decrease the OCLN-4YF mutant levels (Fig. 7A). In the presence of CHX and H_2_O_2_, the decrease of OCLN, but not OCLN-4YF, become evident, indicating that the decrease of OCLN was mediated by protein phosphorylation and degradation (Fig. 7B). Consistent with this, H_2_O_2_-induced OCLN decrease could be prevented by the proteosome inhibitor MG132 (Fig. 7C). We also found that OCLN is heavily ubiquitinated upon H_2_O_2_ treatment, while OCLN-4YF was refractory to H_2_O_2_-induced ubiquitination (Fig. 7D). Our data implicated that Tyr phosphorylation on OCLN was involved in targeting the modified protein for ubiquitination and degradation.

**Fig. 8:**
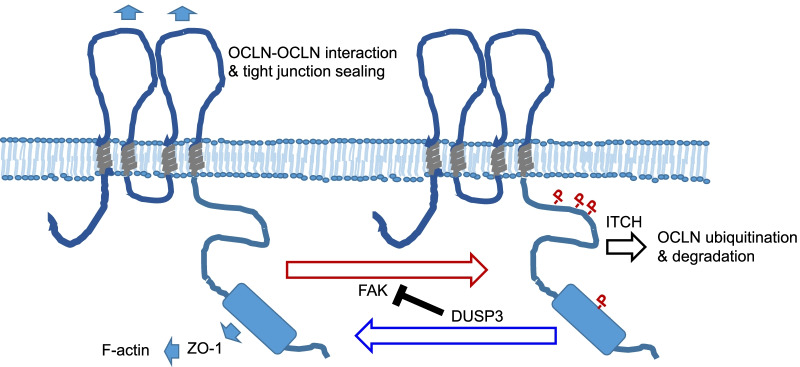
Schematic illustration of DUSP3 regulation on phosphorylation-mediated degradation of OCLN. DUSP3 regulates OCLN through removing OCLN tyrosine phosphorylation directly and through suppressing FAK as well as FAK-OCLN interaction. Phosphorylated OCLN is ubiquitinated by ITCH and degraded through the proteasome pathway

OCLN ubiquitination is mediated by an E3 ubiquitin-protein ligase, ITCH [44]. To test our hypothesis, we examined the interaction between wild type and OCLN-4YF mutant with ITCH. Indeed, OCLN had an increased interaction with its E3 ligase ITCH upon H_2_O_2_ treatment. In contrast, there was no inducible interaction between OCLN-4YF mutant and ITCH despite their interaction in untreated cells was preserved (Fig. 7E). Additionally, we found that while OCLN had an increased interaction with FAK upon H_2_O_2_ treatment, OCLN-4YF mutant did not (Fig. 7F). Our data suggested that tyrosine residues at the C-terminus of OCLN might not only be the phosphorylation sites targeted by FAK, they might also participate in the recruitment of FAK into the protein complex under oxidative stress. Using the H1299-DUSP3_TR_ cell system, we found that DUSP3 expression decreased the interaction between OCLN and FAK and that between OCLN and ITCH, whereas the expression of DUSP3-CS mutant caused the opposite phenomena (Fig. 7G). Taken together, our data suggested that DUSP3 and FAK had counteracting effects on regulating OCLN ubiquitination and degradation (Fig. 8).

## Discussion

Decreasing DUSP3 expression is observed in several type of cancers, including lung cancer [18, 45, 46]. As a phosphatase of EGFR, DUSP3 preferentially dephosphorylates Tyr992 of EGFR and suppresses downstream phospholipase C γ/PKC signaling [18]. *EGFR* mutation at the kinase domain, which leads to constitutional EGFR activation, is one of the major genetic defects found in lung adenocarcinoma [47–49]. Low DUSP3 expression is also observed in lung squamous cell carcinoma, which does not have *EGFR* mutation [18, 50]. Therefore, DUSP3 reduction might be more than a subordinate event that augments the transformation advantage provided by EGFR. It is likely that loss of DUSP3 contributes more than enhancing receptor tyrosine kinase (RTK) signaling during cancer progression. DUSP3 also dephosphorylates FAK, and DUSP3 deficiency leads to increasing phospho-FAK levels at the focal adhesions [30]. In this study, we found that TJ was defective in DUSP3-null cells, suggesting a novel mechanism by which DUSP3 deficiency participates in cancer progression.

The importance of TJ in maintaining barrier functions of epithelial tissues and the involvement of TJ failure in carcinoma progression have been extensively studied [1–4]. In epithelial cells, TJ is regulated by various signaling pathways and by distinct stimuli [1, 2]. This study improves the understanding of TJ regulation by revealing the presence of DUSP3 in the ever-expanding TJ complex. The most evident change in TJ of DUSP3-deficient cells was the decrease of ZO-1. Although our data showed the close proximity between ZO-1 and DUSP3 and suggested that half-life of ZO-1 protein is shorter in DUSP3−/− cells; however, we did not have evidence to support a direct effect of DUSP3 on ZO-1 phosphorylation. Inhibition of FAK, the known DUSP3 substrate, actually further decreased the levels of ZO-1 in lung epithelial cells. These data suggested that the mechanism causing ZO-1 downregulation in DUSP3-deficient cells may involve multiple factors yet to be determined.

In contrast, phosphorylation level of OCLN was increased in DUSP3−/− cells and our collective data supported that OCLN was a direct substrate of DUSP3. According to the collective proteomic research data in PSP, OCLN is phosphorylated on multiple residues at the carboxyl terminus. Because of the preference of DUSP3 against phospho-Tyr, we have focused on studying OCLN Tyr phosphorylation. Among the Tyr residues of OCLN, only few of them have been characterized for their functions. Phosphorylations of OCLN on Tyr 398 and 402 are mediated by Src and is involved in the dissociation between ZO-1 and pOCLN [34]. However, our data indicated that Tyrs 398 and 402 were minor Tyr phosphorylation sites. This result was consistent with the entries in the PSP proteomic database. In contrast, we found that mutation on Y287, 315, 325, or 443 residue all caused more evident decrease of Tyr phosphorylation than that in OCLN-Y398/402 mutant. This disagreement may be due to that the truncated OCLN protein (chicken OCLN a.a. 358–504 and human OCLN a.a. 378–522) used to identify Y398/402 phosphorylation by Src in vitro does not contain most of major Tyr phosphorylation sites [34]. Nevertheless, the universal and unproportioned decreases of Tyr phosphorylation caused by any single mutation on Y287, 315, 325, or 443 residue were unexpected. One possible explanation was that the mutations of these Tyr residues not only abolished the phosphorylation event but also decreased the presence of effecting PTKs in the TJ complex. The decreased OCLN-FAK interaction upon oxidative stress in the OCLN-4YF mutant was consistent with this speculation.

Interaction between FAK and TJ proteins (OCLN and ZO-1) has been reported previously [51, 52]. FAK is a substrate of DUSP3 and our data indicated that FAK phosphorylated OCLN. It was possible that DUSP3 decreased OCLN phosphorylation via inhibiting FAK. Additionally, the result of in vitro phosphatase assays showed that DUSP3 dephosphorylated OCLN directly. DUSP3 completely removed Tyr phosphorylations from OCLN; nevertheless, it was still reasonable to speculate that DUSP3 had an order of preference among all the pTyr residues of OCLN protein. Under the same assay condition, DUSP3 fails to remove phosphorylation on EGFR Tyr 1045 and 1173 residues [18, 30]; therefore, it is very likely that OCLN is a genuine substrate targeted by DUSP3. It should be noted that the expression of DUSP3 decreased FAK-OCLN interaction under oxidative stress (Fig. 7G). OCLN-4YF mutant also has decreased interaction with FAK under oxidative stress (Fig. 7F). DUSP3 regulates FAK phosphorylation by Src under the UV-induced stress condition [53]. Although our data did not support a role for DUSP3 in regulating Src (Fig. 3B), DUSP3 might target both FAK and OCLN under oxidative stresses. Additionally, OCLN-4YF mutant did not have increasing interaction with ITCH, the OCLN E3 ubiquitin ligase (Fig. 7E) and was refractory to ubiquitination and degradation under oxidative stress (Fig. 7D). Our data indicated that DUSP3 may regulate OCLN through a two-tier mechanisms (Fig. 8). The activation of FAK and loss of DUSP3 both can lead to increased ubiquitination and degradation of OCLN and TJ disruption. Activation of FAK and loss of DUSP3 could be cellular responses to oxidative stress or other environmental stimuli, but they could also be events associated with cell transformation and oncogenesis.

Despite the multiple effects of DUSP3 seemed to support a tumor-suppressive role, we did not observe lung cancer (or other tumors) formation in DUSP3−/− mice during the life span examined (up to 18 months). Combination of Tg EGFR-Del mutant and DUSP3 deficiency did facilitate LAC progression. This was consistent with a recent report that DUSP3−/− mice did not develop hepatocellular carcinoma unless combined with the treatment of high-fat diet or a liver carcinogen diethyl nitrosamine (DEN) [54]. It should be noted that increase of DUSP3 expression is reported in several types of cancer [55, 56]. Suppression of DUSP3 has been shown to associate with attenuation of DNA repair ability [57]. Recent reports showing that knockdown of DUSP3 expression is associated with the hyperphosphorylation of a nucleoplasmin protein NPM and G2/M cell cycle arrest [58, 59]. DUSP3 also appears to be an NPM phosphatase and regulates downstream HDM2-p53 interaction and genomic stability upon UV irradiation [58]. Interestingly, we also observed DNA hyperploidy and hypoploidy of DUSP3-deficient cells in comparison to wild type cells (Additional file 1: Fig. S2, Additional file 1), supporting the potential role of DUSP3 in maintaining genome stability. The role of DUSP3 and related molecular mechanisms involved in cancer development deserve more investigations. This study has provided novel insights into the role of DUSP3 in regulating the cell–cell junction in epithelial tissues.

## Conclusions

Our results revealed that TJs were defective in DUSP3-deficient epithelial cells. DUSP3 deficiency was associated with worse outcomes of lung adenocarcinoma in experimental animals and in patients. We identified that OCLN was a direct substrate of DUSP3. DUSP3 regulated OCLN ubiquitination and degradation through decreasing OCLN tyrosine phosphorylation directly or through suppressing focal adhesion kinase. Taken together, DUSP3 is an important TJ regulatory protein and its decrease may be involved in progression of epithelial cancers.

## Supplementary Information

**Additional file 1: Figure S1.** DUSP3-deficient cells have defective tight junction. **Figure S2.** DUSP3-deficient cells have different cell cycle distribution patterns. **Figure S3.** Histological sections of lung tissues from DUSP3 +/+ and DUSP3 −/− mice

## Data Availability

The data presented in the study are included in the article and the additional file. The analysis of correlation between DUSP3 expression and survival of LAC patients is available at the Kaplan Meier Plotter (http://kmplot.com/analysis/index.php?p = service&cancer=pancancer_rnaseq).

## Abbreviations

CHX: Cycloheximide
DTT: Dithiothreitol
DUSP: Dual-specificity phosphatase
EGFR: Epidermal growth factor receptor
EMT: Epithelial-mesenchymal transition
FAK: Focal adhesion kinase
IF: Immunofluorescence
IP: Immunoprecipitation
LAC: Lung adenocarcinoma
OCLN: Occludin
PLA: Proximity ligation assay
PSP: PhosphoSitePlus
PTK: Protein tyrosine kinase
TEER: Trans-epithelial electric resistance
TJ: Tight junction
Tyr/Y: Tyrosine
ZO-1: Zonula occludens-1

## References

[1] Citi S, Guerrera D, Spadaro D, Shah J (2014). Epithelial junctions and Rho family GTPases: the zonular signalosome. Small GTPase.

[2] Brune K, Frank J, Schwingshackl A, Finigan J, Sidhaye VK (2015). Pulmonary epithelial barrier function: some new players and mechanisms. Am J Physiol Lung Cell Mol Physiol.

[3] Yu Y, Elble RC (2016). Homeostatic signaling by cell-cell junctions and its dysregulation during cancer progression. J Clin Med.

[4] Martin TA (2014). The role of tight junctions in cancer metastasis. Seminar Cell Dev Biol.

[5] Cummins PM (2012). Occludin: one protein, many forms. Mol Cell Biol.

[6] Mayor R, Etienne-Manneville S (2015). The front and rear of collective cell migration. Nat Rev Mol Cell Biol..

[7] Alonso A, Sasin J, Bottini N, Friedberg I, Friedberg I, Osterman A (2004). Protein tyrosine phosphatases in the human genome. Cell.

[8] Huang C-Y, Tan T-H (2012). DUSPs, to MAP kinases and beyond. Cell & Biosci.

[9] Chen H-F, Chuang H-C, Tan TH (2019). Regulation of dual-specificity phosphatase (DUSP) ubiquitination and protein stability. Int J Mol Sci.

[10] Chen AJ, Zhou G, Juan T, Colicos SM, Cannon JP, Cabriera-Hansen M (2002). The dual specificity JKAP specifically activates the c-Jun N-terminal kinase pathway. J Biol Chem.

[11] Hood KL, Tobin JF, Yoon C (2002). Identification and characterization of two novel low-molecular-weight dual specificity phosphatases. Biochem Biophys Res Commun.

[12] Niwa R, Nagata-Ohashi K, Takeichi M, Mizuno K, Uemura T (2002). Control of actin reorganization by Slingshot, a family of phosphatases that dephosphorylates ADF/Coffilin. Cell.

[13] Wang JY, Lin C-H, Yang C-H, Tan TH, Chen YR (2006). Biochemical and biological characterization of a neuroendocrine-associated phosphatase. J Neurochem.

[14] Lee JY, Yun JH, Lee J, Choi C, Kim JH (2015). Blockade of dual-specificity phosphatase 28 decreases chemoresistance and migration in human pancreatic cancer cells. Sci Rep.

[15] Zhao BM, Keasey SL, Tropea JE, Lountos GT, Dyas BK, Cherry S (2015). Phosphotyrosine substrate sequence motifs for dual specificity phosphatases. PLoS ONE.

[16] Hoyt R, Zhu W, Cerignoli F, Alonso A, Mustelin T, David M (2007). Selective tyrosine dephosphorylation of interferon-activated nuclear STAT5 by the VHR phosphatase. J Immunol..

[17] Li J-P, Fu Y-N, Chen Y-R, Tan T-H (2010). JNK pathway-associated phosphatase dephosphorylates focal adhesion kinase and suppresses cell migration. J Biol Chem.

[18] Wang J-Y, Yeh C-L, Chou H-C, Yang C-H, Fu Y-N, Chen Y-T (2011). Vaccinia H1-related phosphatase is a phosphatase of ErbB receptors and is down-regulated in non-small cell lung cancer. J Biol Chem.

[19] Li J-P, Yang C-Y, Chuang H-C, Lan J-L, Chen D-Y, Chen Y-M (2014). The phosphatase JKAP/DUSP22 inhibits T-cell receptor signalling and autoimmunity by inactivating Lck. Nat Comm.

[20] Gallegos LL, Ng MR, Sowa ME, Selfors LM, White A, Zervantonakis IK (2016). A protein interaction map for cell cell adhesion regulators identifies DUSP23 as a novel phosphatase for β-catenin. Sci Rep.

[21] Ishibashi T, Bottaro DP, Chan A, Miki T, Aaronson SA (1992). Expression cloing of a human dual-specificity phosphatase. Proc Natl Acad Sci USA.

[22] Todd JL, Rigas JD, Rafty LA, Denu JM (2002). Dual-specificity protein tyrosine phosphatase VHR down-regulates c-Jun N-terminal kinase (JNK). Oncogene.

[23] Todd JL, Tanner KG, Denu JM (1999). Extracellular regulated kinases (ERK) 1 and ERK 2 are authentic substrates for the dual specificity protein-tyrosine phosphatase VHR. J Biol Chem.

[24] Alonso A, Saxena M, Williams S, Mustellin T (2001). Inhibitory role for dual-specificity phosphatase VHR in T cell antigen receptor and CD28-induced ERK and JNK activation. J Biol Chem.

[25] Zhou B, Wang Z-X, Zhao Y, Brautigan DL, Zhang Z-Y (2002). The specificity of extracellular signal-regulated kinase 2 dephosphorylation by protein phosphatases. J Biol Chem.

[26] Hoyt R, Zhu W, Cerignoli F, Alonso A, Mustelin T, David M (2007). Selective tyrosine dephosphorylation of interferon-activated nuclear STAT5 by the VHR phosphatase. J Immunol.

[27] Amand M, Erpicum C, Bajou K, Cerignoli F, Blacher S, Martin M (2014). DUSP3/VHR is a pro-angiogenic atypical dual-specificity phosphatase. Mol Cancer.

[28] Musumeci L, Kuijpers MJ, Gilio K, Hego A, Theatre E, Maurissen L (2015). Dual-specificity phosphatase 3 deficiency or inhibition limits platelet activation and arterial thrombosis. Circulation.

[29] Singh P, Dejager L, Amand M, Theatre E, Vandereyken M, Zurashvili T (2015). DUSP3 genetic deletion confers M2-like macrophage-dependent tolerance to septic shock. J Immunol.

[30] Chen Y-R, Chou H-C, Yang C-H, Chen H-Y, Liu Y-W, Lin T-Y (2017). Deficiency in VHR/DUSP3, a suppressor of focal adhesion kinase, reveals its role in regulating cell adhesion and migration. Oncogene.

[31] Yang C-H, Chou H-C, Fu Y-N, Yeh CLC, Chang I-C, Liu K-J (2015). EGFR over-expression in non-small cell lung cancers harboring EGFR mutations is associated with marked down-regulation of CD82. BBA-Mol Basis Dis..

[32] Hornbeck PV, Zhang B, Murray B, Kornhauser JM, Latham VS, PhosphoSitePlus E (2014). mutations, PTMs and recalibrations. Nucleic Acids Res.

[33] PhosphoSitePlus. https://www.phosphosite.org/proteinAction.action?id=7592&showAllSites=true. Accessed 30 Dec 2020.

[34] Elias BCS, Seth A, Giorgianni F, Kale G, Shen L, Turner JRN (2009). Phosphorylation of Tyr-398 and Tyr-402 in occludin prevents its interaction with ZO-1 and destabilizes its assembly at the tight junctions. J Biol Chem..

[35] Fredriksson S, Gullberg M, Jarvius J, Olsson C, Pietras K, Gustafsdottir SM (2002). Protein detection using proximity-dependent DNA ligation assays. Nat Biotechnol.

[36] Kumar G (2018). A simple method for detecting phosphorylation of proteins by using Zn^2+^-phos-tag SDS-PAGE at neutral pH. Methods Mol Biol.

[37] Nagy A, Munkacsy G, Gyorffy B (2021). Pancancer survival analysis of cancer hallmark genes. Sci Rep.

[38] Kaplan Meier plotter. http://kmplot.com/analysis/index.php?p = service&cancer=pancancer_rnaseq. Accessed 24 Jul 2021.

[39] Denu JM, Zhou G, Wu L, Zhao R, Yuvaniyama J, Saper MA (1995). The purification and characterization of a human dual-specific protein tyrosine phosphatase. J Biol Chem.

[40] Schumacher MA, Todd JL, Rice AE, Tanner KG, Denu JM (2002). Structural basis for the recognition of a bisphosphorylated MAP kinase peptide by human VHR protein phosphatase. Biochemistry.

[41] Xiao Q, Luechapanichkul R, Zhai Y, Pei D (2013). Specificity profiling of protein phosphatases toward phosphoseryl and phosphothreonyl peptides. J Am Chem Soc.

[42] Rao R (2009). Occludin phosphorylation in regulation of epithelial tight junctions. Ann NY Acad Sci.

[43] Wachtel M, Frei K, Ehler E, Fontana A, Winterhalter K, Gloor SM (1999). Occludin proteolysis and increased permeability in endothelial cells through tyrosine phosphatase inhibition. J Cell Sci.

[44] Traweger A, Fang D, Liu Y-C, Stelzhammer W, Krizbai I, Fresser F (2002). The tight junction-specific protein occludin is a functional target of the E3 ubiquitin-protein ligase Itch. J Biol Chem.

[45] Hao L, ElShamy WM (2007). BRCA1-IRIS activates cyclin D1 expression in breast cancer cells by downregulating the JNK phosphatase DUSP3/VHR. Int J Cancer.

[46] Wagner KW, Alam H, Dhar SS, Giri U, Li N, Wei Y (2013). KDM2A promotes lung tumorigenesis by epigenetically enhancing ERK1/2 signaling. J Clin Invest.

[47] Huang S-F, Liu H-P, Li L-H, Ku Y-C, Fu Y-N, Tsai H-Y (2004). High frequency of epidermal growth factor receptor mutations with complex patterns in non-small cell lung cancer related to gefitinib responsiveness in Taiwan. Clin Cancer Res.

[48] Shigematsu HLL, Takahashi T, Nomura M, Suzuki M, Wistuba II, Fong KM, Lee H, Toyooka S, Shimizu N, Fujisawa T, Feng Z, Roth JA, Herz J, Minna JD, Gazdar AF (2005). Clinical and biological features associated with epidermal growth factor receptor gene mutations in lung cancers. J Natl Cancer Inst.

[49] Chen Y-R, Fu Y-N, Lin C-H, Yang S-T, Hu S-F, Chen Y-T (2006). Distinctive activation patterns in constitutively active and gefitinib-sensitive EGFR mutants. Oncogene.

[50] Liu H-P, Wu H-DI, Chang JW-C, Wu Y-C, Yang H-Y, Chen Y-T, et al. Prognostic implications of epidermal growth factor receptor and KRAS gene mutations and epidermal growth factor receptor gene copy numbers in patients with surgically resectable non-small cell lung cancer in Taiwan. J Thorac Oncol. 2010;5:1175–84. 10.1097/JTO.0b013e3181e2f4d6 20559151

[51] Siu ER, Wong EWP, Mruk DD, Porto CS, Cheng CY (2009). Focal adhesion kinase is a blood–testis barrier regulator. Proc Natl Acad Sci USA.

[52] Dorfel MJ, Huber O (2012). Modulation of tight junction structure and function by kinases and phosphatases targeting occludin. J Biomed Biotechnol.

[53] Pereira NR, Russo LC, Forti FL (2021). UV radiation-induced impairment of cellular morphology and motility is enhanced by DUSP3/VHR loss and FAK activation. Cell Biochem Biophys.

[54] Jacques S, Arjomand A, Perée H, Collins P, Mayer A, Lavergne A (2021). Dual-specificity phosphatase 3 deletion promotes obesity, non-alcoholic steatohepatitis and hepatocellular carcinoma. Sci Rep.

[55] Henkens R, Delvenne P, Arafa M, Moutschen M, Zeddou M, Tautz L (2008). Cervix carcinoma is associated with an up-regulation and nuclear localization of the dual-specificity protein phosphatase VHR. BMC Cancer.

[56] Arnoldussen YJ, Lorenzo PI, Pretorius ME, Waehre H, Risberg B, Maelandsmo GM (2008). The mitogen-activated protein kinase phosphatase Vaccinia H1–related protein inhibits apoptosis in prostate cancer cells and is overexpressed in prostate cancer. Cancer Res.

[57] Torres TEP, Russo LC, Santos A, Marques GR, Magalhaes YT, Tabassum S (2017). Loss of DUSP3 activity radiosensitizes human tumor cell lines via attenuation of DNA repair pathways. Biochim Biophys Acta Gen Subj.

[58] Russo LC, Ferruzo PYM, Forti F (2021). Nucleophosmin protein dephosphorylation by DUSP3 is a fine-tuning regulator of p53 signaling to maintain genomic stability. Front Cell Dev Biol.

[59] Russo LC, Farias JO, Forti FL (2020). DUSP3 maintains genomic stability and cell proliferation by modulating NER pathway and cell cycle regulatory proteins. Cell Cycle.

