# Supplementary material for: DUSP3 regulates phosphorylation-mediated degradation of occludin and is required for maintaining epithelial tight junction
Source: J Biomed Sci. 2022 Jun 15;29:40. doi: 10.1186/s12929-022-00826-x (PMC9199239; doi:10.1186/s12929-022-00826-x)
Supplement: Supplementary file 1 — Additional file 1: Figure S1. DUSP3-deficient cells have defective tight junction. Figure S2. DUSP3-deficient cells have different cell cycle distribution patterns. Figure S3. Histological sections of lung tissues from DUSP3 +/+ and DUSP3 −/− mice [file 12929_2022_826_MOESM1_ESM.ppt]

## Slide 1
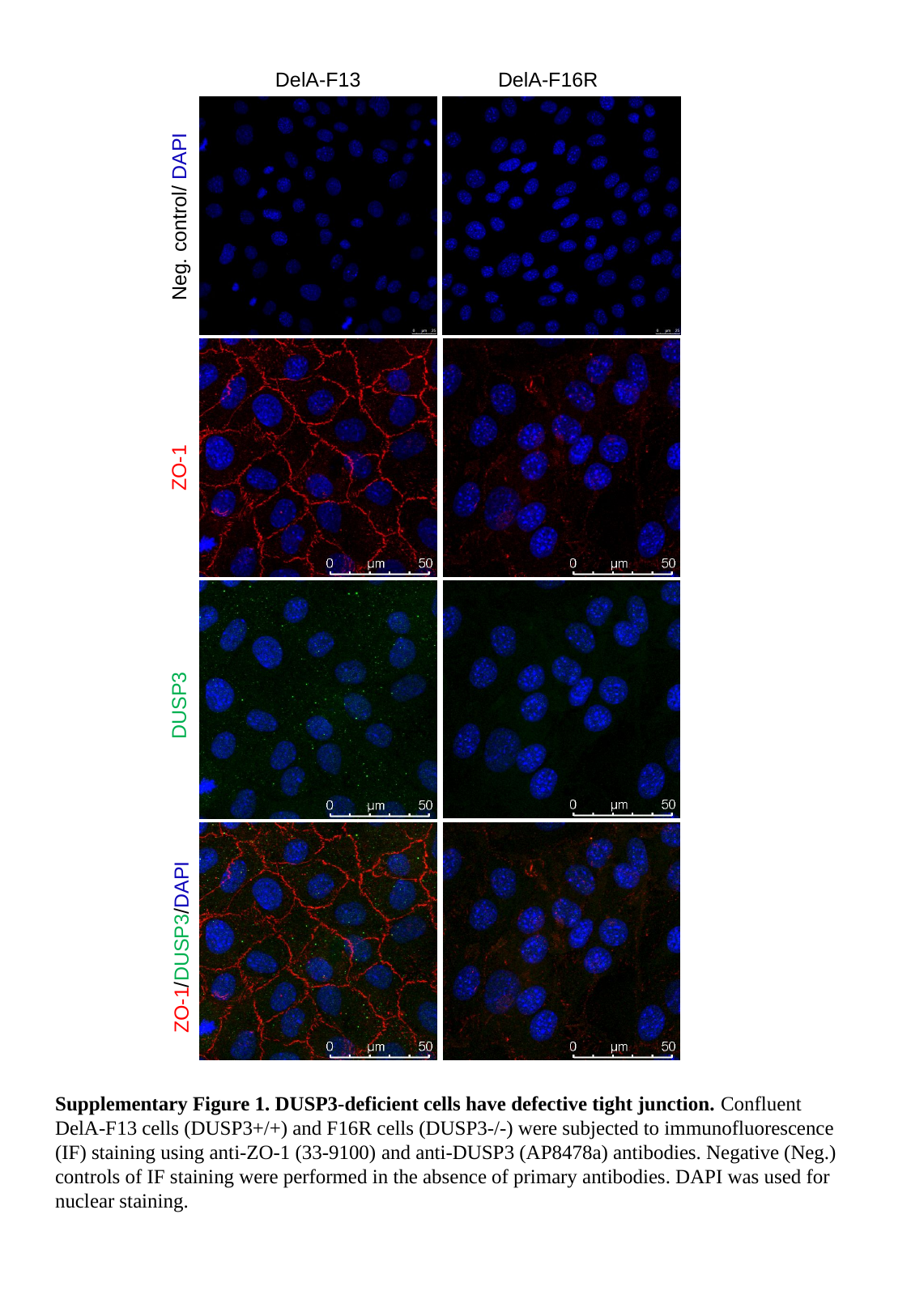

DelA-F13
DelA-F16R
Neg. control/ DAPI
ZO-1
DUSP3
ZO-1/DUSP3/DAPI
Supplementary Figure 1. DUSP3-deficient cells have defective tight junction. Confluent DelA-F13 cells (DUSP3+/+) and F16R cells (DUSP3-/-) were subjected to immunofluorescence (IF) staining using anti-ZO-1 (33-9100) and anti-DUSP3 (AP8478a) antibodies. Negative (Neg.) controls of IF staining were performed in the absence of primary antibodies. DAPI was used for nuclear staining.

## Slide 2
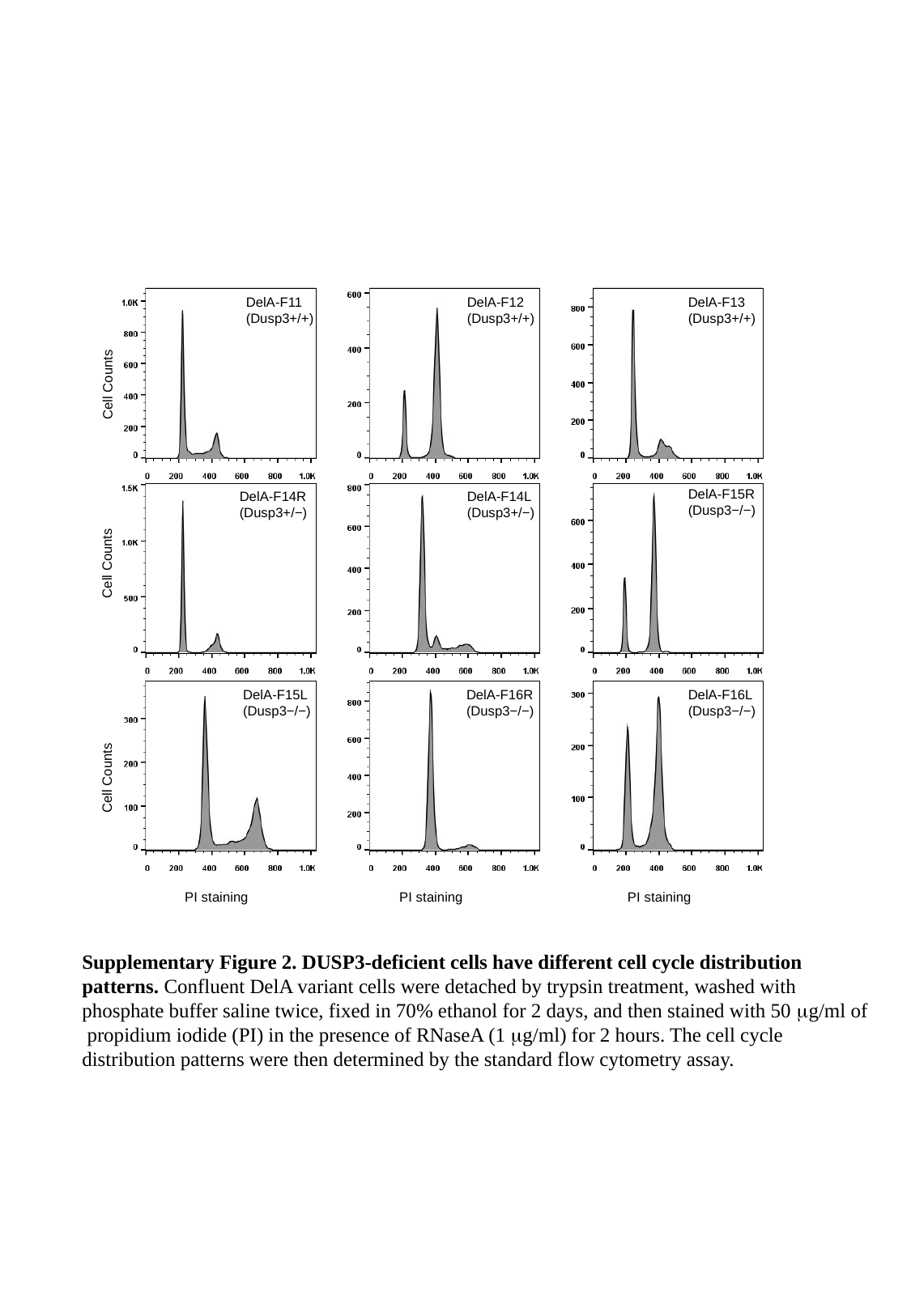

DelA-F11
(Dusp3+/+)
DelA-F13
(Dusp3+/+)
DelA-F12
(Dusp3+/+)
Cell Counts
DelA-F15R
(Dusp3−/−)
DelA-F14R
(Dusp3+/−)
DelA-F14L
(Dusp3+/−)
Cell Counts
DelA-F15L
(Dusp3−/−)
DelA-F16R
(Dusp3−/−)
DelA-F16L
(Dusp3−/−)
Cell Counts
PI staining
PI staining
PI staining
Supplementary Figure 2. DUSP3-deficient cells have different cell cycle distribution patterns. Confluent DelA variant cells were detached by trypsin treatment, washed with phosphate buffer saline twice, fixed in 70% ethanol for 2 days, and then stained with 50 μg/ml of propidium iodide (PI) in the presence of RNaseA (1 μg/ml) for 2 hours. The cell cycle distribution patterns were then determined by the standard flow cytometry assay.

## Slide 3
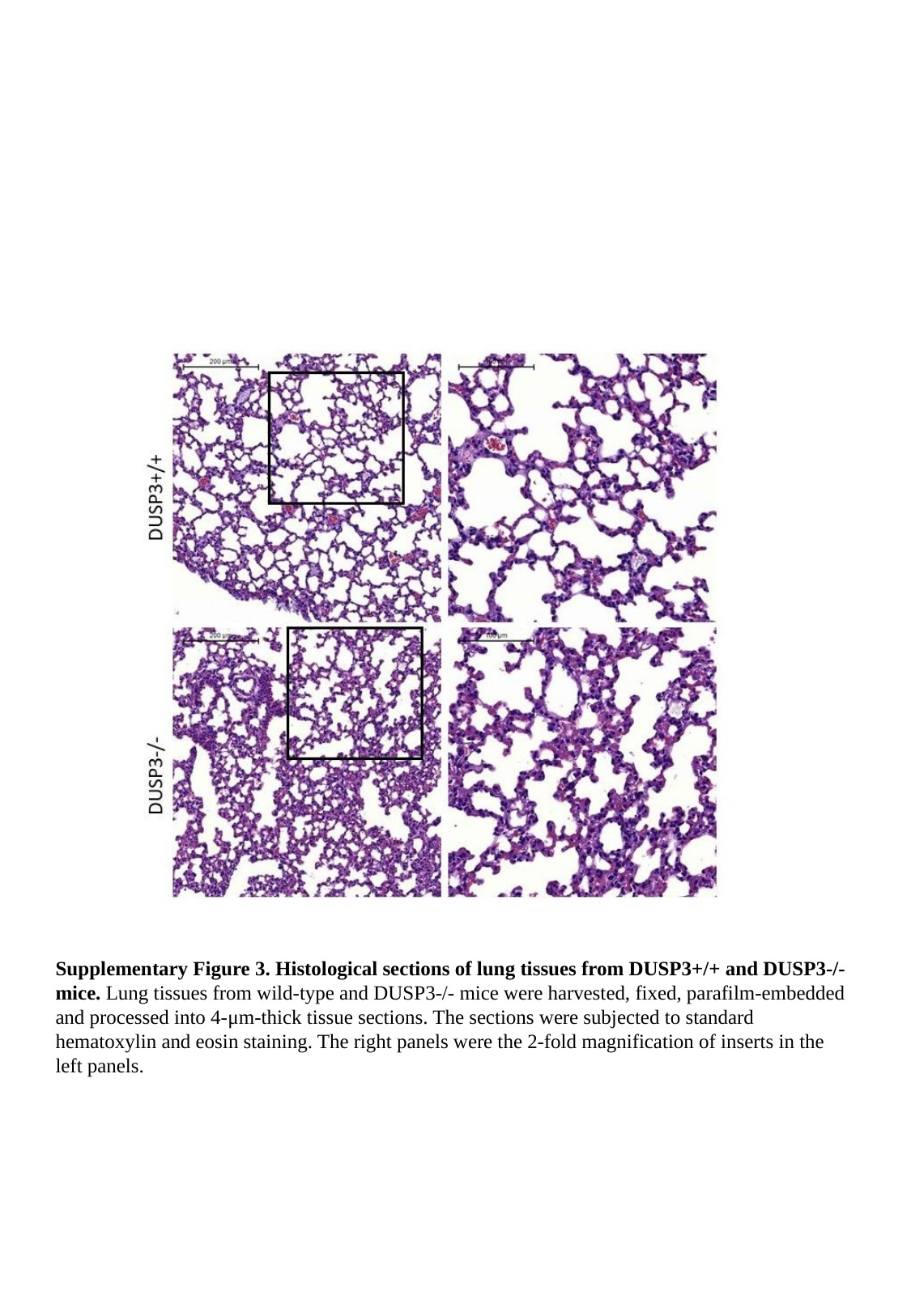

Supplementary Figure 3. Histological sections of lung tissues from DUSP3+/+ and DUSP3-/- mice. Lung tissues from wild-type and DUSP3-/- mice were harvested, fixed, parafilm-embedded and processed into 4-μm-thick tissue sections. The sections were subjected to standard hematoxylin and eosin staining. The right panels were the 2-fold magnification of inserts in the left panels.
